# Understanding communication between patients and healthcare professionals regarding comprehensive biomarker testing in precision oncology: A scoping review

**DOI:** 10.1002/cam4.6913

**Published:** 2024-01-31

**Authors:** Theresia Pichler, Friederike Mumm, Navdeep Dehar, Erin Dickman, Celia Díez de Los Ríos de la Serna, Andreas Dinkel, Kathrin Heinrich, Merel Hennink, Anndra D. Parviainen, Vincent Raske, Nicole Wicki, Amy C. Moore

**Affiliations:** ^1^ Department of Internal Medicine III, University Hospital LMU Munich Munich Germany; ^2^ Comprehensive Cancer Center Munich LMU (CCC Munich) Munich Germany; ^3^ Department of Medical Oncology Queen's University Kingston Ontario Canada; ^4^ Oncology Nursing Society Pittsburgh Pennsylvania USA; ^5^ European Oncology Nursing Society Brussels Belgium; ^6^ Faculty of Medicine and Health Sciences, School of Nursing Barcelona University Barcelona Catalonia Spain; ^7^ Department of Psychosomatic Medicine and Psychotherapy, Klinikum rechts der Isar, School of Medicine and Health Technical University of Munich Munich Germany; ^8^ Comprehensive Cancer Center Munich TUM (CCC Munich) Munich Germany; ^9^ Stichting Merels Wereld Groningen The Netherlands; ^10^ Department of Nursing Science, Faculty of Health Sciences University of Eastern Finland Kuopio Finland; ^11^ The Synergist Brussels Belgium; ^12^ LUNGevity Foundation Chicago Illinois USA

**Keywords:** biomarkers, communication, decision making, shared, neoplasms, physician‐patient relations, precision medicine, psycho‐oncology

## Abstract

**Background:**

Precision oncology, using comprehensive biomarker testing (cBT) to inform individual cancer diagnosis, prognosis and treatment, includes increasingly complex technology and clinical data sets. People impacted by cancer (patients and caregivers) and healthcare professionals (HCPs) face distinct challenges in navigating the cBT and personalized treatment landscape. This review summarizes evidence regarding cBT‐related communication between people impacted by cancer and HCPs and identifies important avenues for future research in precision oncology.

**Methods:**

A scoping review was conducted using records published in PubMed during January 2017–August 2022, focusing on the breadth of topics on patient‐HCP communication and knowledge resources used by HCPs as guidance in cBT‐related communication. Data were extracted from records meeting inclusion criteria, and findings were summarized according to main topics.

**Results:**

The search identified 287 unique records and data were extracted from 42 records, including nine from expert input. Most records originated from the United States included patients with different types of cancer, and oncologists were the main HCPs. Patients' motivation for undergoing cBT and receiving results was generally high in different settings. However, patients' understanding of cBT‐related concepts was limited, and their knowledge and information preferences changed based on cBT implications and significance to family members. HCPs were valued by patients as a trusted source of information. Limited evidence was available on HCPs' information‐seeking behavior and factors influencing cBT‐related knowledge and confidence, often self‐reported as insufficient.

**Conclusions:**

Patient education by knowledgeable and confident HCPs, information management and a caring patient‐HCP relationship communicating continuity of care regardless of cBT results are crucial to empower patients and shared decision‐making in precision oncology. More data on the process and structure of cBT‐related communication, distinction between and characterization of different timepoints of patient‐HCP interactions are needed.

## 1 INTRODUCTION

Precision oncology has become the cornerstone of modern cancer care. In this review, precision oncology is defined as biomarker‐driven oncology, where molecular information from cancer cell profiling is used to guide diagnosis, prognosis, and treatment.^1^, ^2^, ^3^ Since current literature uses variable terms to refer to profiling of patients' tumor samples, here we use comprehensive biomarker testing (cBT) as a consensus term for different profiling assays.

cBT encompasses methods to detect various genomic alterations, such as driver variants (e.g., mutations), chromosome rearrangements, and changes in gene expression profiles.^4^, ^5^

In the last decade, fast‐evolving medical technologies, such as the expansion of high‐throughput next‐generation sequencing, have increased the availability of cBT and thus hope for improved cancer treatment.^6^, ^7^ The inherent paradigm shift to histology‐agnostic, biomarker‐directed treatment has profoundly changed the interplay of research and clinical practice and the collaboration of different professions within the medical field.^8^, ^9^

This paradigm shift has influenced patient management, whereby standard practices for evidence generation (e.g., registration trials and integration of real‐world studies) blur the lines between research and clinical practice.^8^ This raises considerable ethical, legal, and procedural questions, given the different principles of the two fields.^10^ Consequently, this approach requires continuous adjustments of regulations and structures (e.g., concerning the large data outputs), increasing the complexity of the modern precision oncology field.

Such complexity calls for a new dimension of interdisciplinary team cooperation to ensure state‐of‐the‐art cBT‐guided precision oncology.^7^ This cooperation is ideally organized in molecular tumor boards, consisting of oncologists, pathologists, geneticists, and molecular biology specialists, among others.^7^, ^11^

Further, this complexity challenges both the healthcare professionals' (HCPs) and patients' understanding of precision oncology aspects (e.g., individual benefit of cBT and the targeted treatment for the patient).^7^, ^11^, ^12^ Steady progress and remarkable achievements have been made with respect to cBT‐informed treatments^8^, ^13^; however, a therapeutic benefit of such treatments is still not always guaranteed.^8^, ^14^ This is often in strong contrast to the high expectations of patients and their families,^15^, ^16^ which could be elevated by a lack of knowledge that potentially also influences their decision‐making toward cBT.^8^, ^17^, ^18^ Patients, but also HCPs, face uncertainty regarding the choice of cBT approaches and targets, optimal timing to conduct cBT and the treatments that may be available to patients.^18^, ^19^, ^20^, ^21^, ^22^ Furthermore, shared decision‐making, whereby HCPs and patients collaboratively explore available care options and decide the best course of action based on benefits, harms and preferences, is an emerging practice in patient care.^23^, ^24^, ^25^ Considering the described developments and associated uncertainties, significant challenges surround cBT‐related communication and patient management in precision oncology. So far, evidence on characteristics of patient‐HCP communication and associated impacts on shared decision‐making and multidisciplinary team cooperation in this field is limited. Therefore, this scoping review aims to characterize these relevant aspects of cBT‐related communication and highlight the roles of HCPs in multidisciplinary teams.

## 2 METHODS

### 2.1 Search strategy and criteria

This was a scoping literature review. PubMed was searched for articles published January 01, 2017–August 26, 2022. Controlled MeSH terms were used where possible and combined with selected keywords. The selected keywords covered terms and synonyms of “oncology,” “comprehensive genomic profiling,” “precision medicine,” “shared decision‐making,” “communication,” “patients,” “healthcare professionals,” “guidelines,” and “support” and were combined into two search strings, to cover the complexity and breadth of the field of interest (Table 1).

**TABLE 1:** Search strings used in PubMed.

Search string	Number of hits in PubMed (filter January 01, 2017–August 26, 2022)
((“Communication”[MeSH Terms] OR “Health Communication”[MeSH Terms] OR “communicat*”[Title] OR “inform*”[Title] OR “disclos*”[Title] OR “discuss*”[Title] OR “interact*”[Title]) AND (“Precision Medicine”[MeSH Terms] OR “High‐Throughput Nucleotide Sequencing”[MeSH Terms] OR “Whole Genome Sequencing”[MeSH Terms] OR “genomic test*”[Title] OR “sequencing”[Title] OR “genomic profiling”[Title]) AND (“Neoplasms”[MeSH Terms] OR “tumor”[Title] OR “cancer”[Title] OR “tumor”[Title] OR “cancer patient*”[Title]) AND (“Therapeutics”[MeSH Terms] OR “Patient Care Management”[MeSH Terms] OR “Decision Making”[MeSH Terms] OR “Clinical Decision‐Making”[MeSH Terms] OR “decision making, shared”[MeSH Terms] OR “treatment decision*”[Title/Abstract] OR “therap*”[Title/Abstract] OR “care management”[Title/Abstract] OR “decision*”[Title/Abstract]))	262
(“Education”[MeSH Terms] OR “Training Support”[MeSH Terms] OR “Staff Development”[MeSH Terms] OR “expectation*”[Title/Abstract] OR “need*”[Title/Abstract] OR “support”[Title/Abstract] OR “resource*”[Title/Abstract] OR “training”[Title/Abstract] OR “educat*”[Title/Abstract] OR guide* [Title/Abstract]) AND (“Health Personnel”[MeSH Terms] OR “Oncologists”[MeSH Terms] OR “physician*”[Title/Abstract] OR “healthcare professional*”[Title/Abstract] OR “healthcare provider*”[Title/Abstract] OR “oncologist*”[Title/Abstract]) AND (“Communication”[MeSH Terms] OR “Health Communication”[MeSH Terms] OR “communicat*”[Title] OR “inform*”[Title] OR “disclos*”[Title] OR “discuss*”[Title]) AND (“Precision Medicine”[MeSH Terms] OR “High‐Throughput Nucleotide Sequencing”[MeSH Terms] OR “Whole Genome Sequencing”[MeSH Terms] OR “genomic test*”[Title/Abstract] OR “sequencing”[Title/Abstract] OR “genomic profiling”[Title/Abstract]) AND (“Neoplasms”[MeSH Terms] OR “tumor”[Title] OR “cancer”[Title] OR “tumor”[Title] OR “cancer patient*”[Title])	39

Records were screened by four independent reviewers in two stages—at title and abstract and at full‐text level. Conflicts of opinion were resolved by consensus opinion between two of the four reviewers.

Records were included if they contained any information related to cBT testing in oncology and pertained to any aspect or phase of cBT‐related communication (pre‐ or post‐testing). Records were excluded if they were out of scope, dealt solely with genetic and germline testing or pediatric oncology. Studies in pediatric populations were excluded because of additional complexity in communication with HCPs facilitated through parents or guardians and potentially different requirements concerning communication and psychosocial support. Expert authors provided several additional records of interest, to contribute important data from different research lenses. The publications from expert referral were screened and selected by authors other than those who made the referrals, and only those publications objectively matching all inclusion criteria were selected.

Data were extracted by three independent reviewers, one of whom unified the findings.

The results were grouped according to common themes and assessed descriptively.

### 2.2 Study protocol registration

Project description was registered on Open Science Framework (https://doi.org/10.17605/OSF.IO/JF54B).

## 3 RESULTS

### 3.1 Search outcomes

**FIGURE 1:**
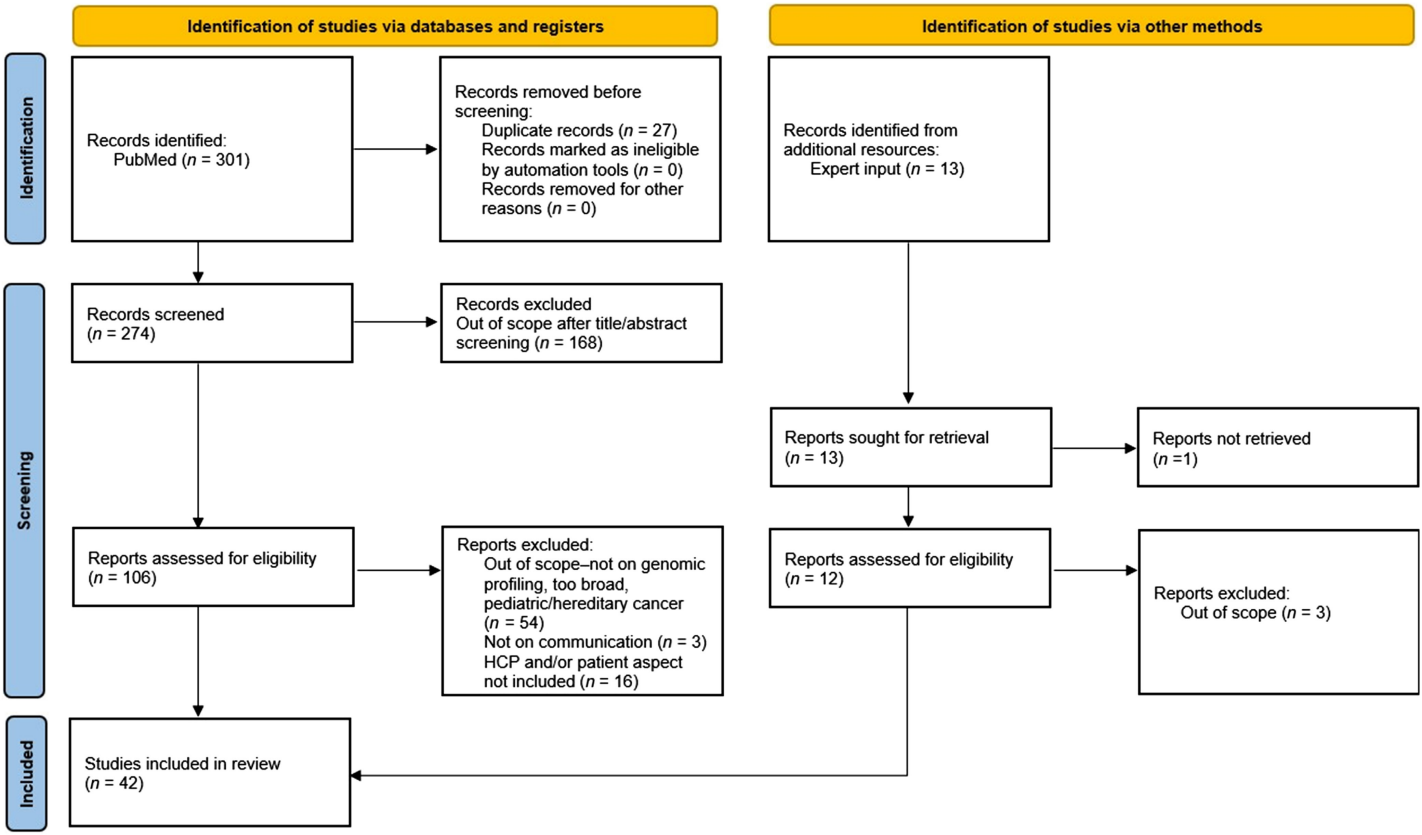
PRISMA flow diagram of identified records. HCP, healthcare professionals; *n*, number of records.

The PubMed search yielded 274 unique records, while 13 records were provided by expert input (Figure 1). After title/abstract and full‐text screening, 33 records from PubMed and nine records from expert input were selected for data extraction and analysis.

Most (20; 47.6%) studies originated from the United States of America (USA), followed by Australia (7; 16.7%) and the Netherlands (4; 9.5%) (Table 2; Table S1). Original studies largely relied on qualitative or combined (qualitative and quantitative) data analysis (18; 42.9%) and, where applicable, focused on oncologists (20; 47.6%). The cancer type was mostly unspecified (20; 47.6%) or patients with different types of cancer were included in the study (13; 31.0%) (Table 2; Table S1). Also, where specified, much of the available data were based on well‐educated patients of European descent (or “non‐Hispanic White”).^17^, ^18^, ^21^, ^22^, ^26^, ^27^, ^28^, ^29^, ^30^, ^31^, ^32^, ^33^ Additionally, evaluation of populations in two review articles showed an underrepresentation of other ethnic and minority groups, which was also supported by author statements in these publications.^34^, ^35^

**TABLE 2:** Summary of study characteristics.

Study parameter	*n* (%)
Total	42 (100)
Study region/country	
AMR	
USA	20 (47.6)^20^, ^21^, ^22^, ^27^, ^28^, ^29^, ^31^, ^32^, ^33^, ^34^, ^42^, ^43^, ^45^, ^47^, ^48^, ^49^, ^50^, ^54^, ^61^, ^80^
Canada	2 (4.8)^18^, ^55^
WPR	
Australia	7 (16.7)^19^, ^30^, ^35^, ^36^, ^37^, ^46^, ^52^
Singapore	1 (2.4)^38^
Japan	1 (2.4)^53^
EUR	
The Netherlands	4 (9.5)^17^, ^26^, ^40^, ^51^
France	3 (7.1)^11^, ^41^, ^44^
Other (Belgium, Germany)	3 (7.1)^7^, ^12^, ^39^
EMR	
Saudi Arabia	1 (2.4)^62^
Study design	
Qualitative	10 (23.8)^12^, ^17^, ^19^, ^20^, ^21^, ^26^, ^36^, ^37^, ^38^, ^53^
Quantitative	7 (16.7)^18^, ^27^, ^28^, ^43^, ^50^, ^51^, ^80^
Combined‐qualitative and quantitative^a^	8 (19.0)^29^, ^30^, ^31^, ^32^, ^33^, ^47^, ^48^, ^49^
Other^b^	17 (40.5)^7^, ^11^, ^22^, ^34^, ^35^, ^39^, ^40^, ^41^, ^42^, ^44^, ^45^, ^46^, ^52^, ^54^, ^55^, ^61^, ^62^
Cancer type	
Lung	2 (4.8)^21^, ^62^
Breast	5 (11.9)^22^, ^28^, ^31^, ^38^, ^43^
Gastro‐intestinal	1 (2.4)^29^
Neuroendocrine tumors	1 (2.4)^51^
Various^c^	13 (31.0)^12^, ^17^, ^18^, ^19^, ^20^, ^26^, ^32^, ^33^, ^36^, ^46^, ^49^, ^50^, ^54^
Not specified	20 (47.6)^7^, ^11^, ^27^, ^30^, ^34^, ^35^, ^37^, ^39^, ^40^, ^41^, ^42^, ^44^, ^45^, ^47^, ^48^, ^52^, ^53^, ^55^, ^61^, ^80^
Specialty of HCPs in focus^d^ ^,^ ^e^	
Oncologists	20 (47.6)^7^, ^18^, ^20^, ^26^, ^30^, ^34^, ^35^, ^39^, ^41^, ^42^, ^44^, ^45^, ^46^, ^47^, ^48^, ^49^, ^50^, ^51^, ^54^, ^55^
Oncology specializations	
Medical oncology	7 (16.7)^18^, ^29^, ^31^, ^43^, ^47^, ^51^, ^54^
Surgical oncology	2 (4.8)^29^, ^47^
Radiation oncology	1 (2.4)^54^
Non‐oncologists	
Genetics specialists	7 (16.7)^7^, ^22^, ^38^, ^41^, ^42^, ^44^, ^55^
(Oncology) nurses	4 (9.5)^21^, ^46^, ^51^, ^62^
Pathologists	4 (9.5)^7^, ^39^, ^53^, ^55^
Biology and bioinformatics specialists	3 (7.1)^7^, ^41^, ^44^
General practitioners/family physicians	2 (4.8)^41^, ^46^
Other^f^	5 (11.9)^22^, ^38^, ^41^, ^53^, ^54^

*Note*: AMR, EMR, EUR, WPR, world regions according to the classification of the World Health Organization as region of the Americas (AMR), Eastern Mediterranean region (EMR), European Region (EUR) and West‐Pacific Region (WPR); HCP, healthcare professional; *n* (%), number and percentage of studies in a given category (note: the percentages do not always add up to 100%); USA, United States of America.

^a^ Combined study design refers to studies that included both quantitative and qualitative elements in their data analysis.

^b^ Studies in this category were literature reviews, descriptive case studies, guidelines, and opinion articles.

^c^ Studies in this category included patients and/or oncologist specializations in several different cancer fields.

^d^ The specialty of HCPs in these studies refers to either experts who were study participants or were mentioned as part of guidelines and recommendations.

^e^ The number of studies does not add up to 42 because some studies in this review did not include HCPs.

^f^ Other specializations included gynecologists, surgeons, palliative care doctors, internal medicine experts, laboratory medicine experts, neurosurgeons, radiologists, research clinicians, medical anthropologists, psychology, and dietetics experts.

### 3.2 Theme 1: Characteristics and processes of patient‐HCP communication

#### 3.2.1 General considerations—Factors influencing cBT‐related communication

Despite heterogeneity in research design and context (i.e., clinical care or precision oncology research), some common themes emerged from the included studies. Patients were motivated to undergo cBT to learn more about their disease, about targeted and experimental treatment options, and to promote cancer research.^30^, ^33^, ^35^, ^36^, ^37^, ^38^, ^39^ Patients were interested in receiving information on different types of cBT results, especially those impacting their own and their children and relatives' health.^17^, ^18^, ^19^, ^28^, ^30^, ^32^, ^33^, ^36^, ^38^, ^40^ However, four studies implied that interest in receiving information on variants of uncertain significance (VUSs) was variable or even absent, depending on the context and understanding of VUS implications.^18^, ^19^, ^28^, ^36^

Several overarching challenges surrounding cBT‐related communication were identified: the patients' general distress due to cancer diagnosis,^12^, ^19^, ^21^, ^37^, ^38^, ^40^, ^41^ limited health literacy and lack of patients' understanding of genomic concepts,^12^, ^17^, ^19^, ^20^, ^37^, ^38^, ^42^, ^43^ uncertain significance of some cBT results,^38^, ^40^, ^44^ a mismatch between high patient expectations and perceived benefit of cBT versus actual testing outcomes^12^, ^20^, ^33^ and a potential lack of actionable alterations and/or therapeutic options based on a patient's genomic profile.^12^, ^20^, ^33^, ^37^, ^41^, ^43^

Also, additional costs and potential insurance issues were challenging for patients interested in undergoing cBT.^19^, ^20^, ^26^, ^35^, ^37^ These concerns of potential health and social discrimination were shared by HCPs surveyed in three other studies.^18^, ^45^, ^46^ Additionally, the included studies often did not specify the timepoints (e.g., days/weeks after diagnosis, pre‐ or post‐surgery or first‐line therapy) and manner (e.g., in‐person or via telephone consultation, type of appointment, used language, time spent) in which HCPs communicated with patients regarding cBT. Finally, it was not always possible to distinguish between pre‐ and post‐testing communication surrounding cBT.

#### 3.2.2 Pre‐testing communication—Considerations pertaining to informed consent forms

We identified eight records focusing on topics related to informed consent forms, of which two focused solely on clinical care,^40^, ^44^ two on clinical care and research,^38^, ^41^ three on the research setting^11^, ^33^, ^42^ and one discussed a broader European legislation context.^39^ Patient autonomy and the possibility to withdraw consent or refuse findings were commonly recognized priorities.^38^, ^40^, ^41^, ^44^

To counter the abovementioned challenges for effective cBT‐related communication, two studies recommended focusing oncologists' efforts to main concepts or only actionable findings in their discussions with patients (see also Table 3).^40^, ^44^ Additionally, one study focusing on minority groups argued that cBT‐related communication must be presented in the context of the patients' educational, ethnic, and cultural background, to maximize cBT uptake among under‐represented populations.^42^

**TABLE 3:** Recommendations for improved ICFs from selected studies.

Study	IC context	Type of findings	IC recommendations
Stoeklé 2018^11^	Research (clinical trials)	Not specified	‐ Collaboration between patients, clinicians, researchers, and industry and even using information technology developments to improve informed consent forms ‐ Carefully formulate the informed consent forms using terms such as “possible,” “conditional,” and “potential” care to ensure that patients fully understand the informed consent form they are requested to sign
Bylstra 2017^38^	Research and clinical care	Not specified	‐ Recommended blanket/broad consent, whereby both HCPs and patients agreed that a trained, neutral person (e.g., a geneticist or another HCP) would be most suitable to provide details on genomic testing
Mamzer 2017^41^	Research and clinical care	Not specified	‐ Collaboration between a cancer patient committee and medical ethics representatives to help formulate suitable informed consent forms
Bunnik 2021^40^	Clinical care	Primary findings, secondary findings, VUSs	‐ Involving medical ethics and genetics experts was preferred in drafting informed consent forms, explaining them to patients and disclosing results ‐ Recommended a layered consent model, where all patients receive essential information in the first layer and, depending on their status, may opt to receive further information in subsequent layers
Pujol 2018^44^	Clinical care (adaptation to research possible)	Secondary findings	‐ Two informed consent forms for disclosure of secondary findings: one at the initial consultation before testing is requested and a second one after primary results are known ‐ Potentially actionable secondary findings may be disclosed to patients' relatives, if authorized by the patient and informed consent forms must allow for sufficient delicacy in this procedure, due to psychological burden that may be imposed on the family
Horgan 2019^39^	Not specified	Not specified	‐ Recommended a dynamic informed consent form, allowing patients to be contacted at a later time if needed

Abbreviations: HCP, healthcare professional; VUS, variants of uncertain significance.

An important aspect of informed consent forms was the distinction between cBT to inform cancer care versus testing within exploratory clinical trials.^11^, ^41^ Three studies recommended different approaches to tailoring informed consent forms to clinical versus research purposes (Table 3).^11^, ^38^, ^41^

#### 3.2.3 Post‐testing and general communication—Additional considerations

Sixteen additional studies focused on general or post‐testing cBT‐related communication in either the clinical care or research context, whereby the distinction between the two was not always clear.^12^, ^17^, ^18^, ^19^, ^20^, ^21^, ^22^, ^26^, ^28^, ^31^, ^32^, ^33^, ^36^, ^37^, ^47^, ^48^

Patients expressed the need for psychosocial support when receiving cBT results^37^ and especially secondary findings as these results may expose personal vulnerabilities, imply discouraging health prospects, and negatively affect their family members.^17^, ^26^

Also, four studies pointed out that patients' motivation to undergo cBT and high expectations for an individualized or curative treatment may be misplaced due to their limited understanding or an exaggerated promise of cBT as presented in the media.^19^, ^20^, ^21^, ^36^

In return, HCPs frequently reported having difficulties explaining cBT‐related uncertainties in a way that patients would understand, especially regarding secondary findings or VUSs.^20^, ^21^, ^45^, ^46^ Two studies described that the oncologists preferred focusing on actionable alterations as an anchor to explain the significance of non‐actionable findings^22^ or presented actionable alterations too optimistically.^21^ Petrillo et al. further found that oncologists avoided discussing prognosis due to uncertain treatment efficacies and survival benefit in lung cancer patients.^21^

### 3.3 Theme 2: Mechanisms to support shared decision‐making in precision oncology

Overall, patients viewed HCPs as the most trusted and impactful information source and valued transparent, empathic, and accessible communication.^12^, ^19^, ^32^, ^35^, ^37^ Some patient groups were more motivated or equipped to participate in shared decision‐making surrounding cBT‐related discussions with their physician. Three studies showed that well informed patients with a high level of genomics knowledge and/or previous genetic testing experience were more interested in cBT‐related aspects.^19^, ^21^, ^28^ High levels of patient‐centered communication and shared decision‐making were reported in three other studies,^19^, ^31^, ^43^ of which two found that patients with higher disease recurrence scores were more likely to discuss treatment‐related topics and be equal partners in the decision‐making processes with the HCPs.^31^, ^43^

Patients' families and caregivers were reported to significantly influence the decision‐making process and cBT uptake.^20^, ^45^, ^46^, ^49^ Oncologists surveyed in two studies faced different challenges involving patients and their families in cBT‐related discussions depending on their practice characteristics.^49^, ^50^ Furthermore, time and resources needed to realize cBT‐related discussions were barriers for both oncologists and patients,^20^ while the aspect of cBT costs as a barrier for patients was recognized in one original USA‐based study and one systematic review.^46^, ^48^ Yabroff et al. found that frequency of cBT cost discussions depended on oncologists' specialization and experience with cBT,^48^ while Vetsch et al. reported on HCPs reluctance to offer or order cBT due to its high out‐of‐pocket costs.^46^

To facilitate effective discussions and cBT‐related shared decision‐making, some studies suggested developing additional information materials, to serve as communication aids for physicians and help patients remember consultation details with minimal effort.^20^, ^27^, ^29^, ^35^, ^43^ However, not all strategies were perceived as beneficial. For instance, some types of information support systems (e.g., video summaries) only partially reduced patients' distress and satisfaction levels compared to standard care.^27^, ^51^ This reduction was not always significant. Also, the preference for video‐assisted methods to convey information was supported by relatively more physicians (41.0%) than patients (12.0%) surveyed by Pinheiro et al.^32^

### 3.4 Theme 3: Perspectives of HCPs

Oncologists reported being aware that having a good relationship with their patients and trustworthiness were key.^20^ The oncologists were worried that their patients may not trust them if they showed they could not clearly explain all cBT‐related uncertainties.^20^ However, it may be challenging for HCPs to communicate with their patients, whose poor overall health and cancer‐related comorbidities may exacerbate the limited comprehension of cBT‐related aspects.^52^, ^53^ Evidence on HCPs' knowledge of cBT‐related concepts, the sources they used to educate themselves and their confidence in conveying cBT‐related concepts to their patients was relatively scarce and often subjective.^20^, ^22^, ^43^, ^45^, ^46^, ^47^, ^49^, ^50^, ^54^ Two large studies from the United States showed that oncologists used different resources to learn about new cBT methods depending on their affiliation (academic/non‐academic) and location (urban/suburban/rural).^49^, ^50^ HCPs' understanding of cBT‐related concepts was sometimes self‐reported as insufficient, and cBT was deemed challenging to understand.^38^, ^45^, ^46^, ^47^ Two studies examined oncologists' self‐reported confidence in genomic profiling expertise and in conveying cBT‐related concepts to patients, but neither could correlate this confidence to higher knowledge or more experience in using genomic profiling tests.^43^, ^47^

Need for support materials and additional (formal) training in cBT‐related concepts was thus recognized as a priority for oncologists and other HCPs facing cancer patients.^7^, ^20^, ^45^, ^46^, ^47^, ^49^, ^53^, ^54^, ^55^ Some materials, guidelines, and educational programs to support HCPs in understanding of cBT, decision‐making, and communication of cBT‐related concepts have already been established^56^, ^57^, ^58^, ^59^, ^60^ (Table 4).

**TABLE 4:** Guidance and support materials for HCPs to improve understanding of cBT‐related concepts.

Study	Type of material	Target audience/scope	Recommendations
Jarvik 2014^57^	CSER Consortium and eMERGE Network recommendations	Clinical research investigators	‐ Actionable genomic results and referral for appropriate clinical follow‐up should be offered to participants, if they have consented to receive the results ‐ Investigators should be prepared to return genomic results. However, investigators are not ethically obliged to actively search for actionable results and incidental findings discovered in the research context ‐ Investigators should recognize that clinical research and medical care are distinct in terms of aims and guiding moral principles
Richards 2015^60^	Multi‐stakeholder workgroup (ACMG, AMP, CAP) expert consensus	HCPs involved in treatment decisions	‐ A new classification of variants in Mendelian disorders into one of five categories (“pathogenic,” “likely pathogenic,” “uncertain significance,” “likely benign,” and “benign”) is recommended, based on criteria using relevant evidence ‐ Clinical molecular genetic testing should be done in a certified laboratory and results should be interpreted by a genetics HCP or an equivalent expert
Harris 2016^56^	ASCO clinical practice guideline	HCPs involved in treatment decisions	‐ Aside from the biomarker status, treatment decisions should also consider disease stage, comorbidities, and patient preferences
Li 2017^58^	AMP, ASCO and CAP expert workgroup guidelines	HCPs involved in treatment decisions	‐ A four‐tiered system is recommended to categorize somatic sequence variations based on their clinical significance: tier I, strong significance; tier II, potential significance; tier III, unknown significance; and tier IV, benign or likely benign variants. Significance of each variant should be regularly re‐evaluated to match the fast evolution of precision oncology ‐ Genomic variants should be reported according to a standard nomenclature, with a clear description of the testing methods and limitations ‐ Clinical recommendations should be in accordance with clinical and histological findings and should be presented concisely
Yip 2019^55^	Multi‐stakeholder committee guideline of Canadian experts	HCPs involved in treatment decisions	‐ NGS guideline developed by a steering committee of pathologists, geneticists, oncologists and genetic counselors ‐ Four‐tiered system for classification of actionability of genomic results proposed similar to Li et al.^50^ ‐ Oncologists must be familiar with the classification of results and nomenclature and be able to correctly interpret (potentially with the help of genetics HCPs or pathologists) the results of genomic profiling ‐ Multidisciplinary teams should agree on a patient's best interest both before and after cBT ‐ Before testing, patients should be informed of the testing limitations and implications, including incidental findings
Doll 2021^61^	DoD MHS expert opinion and recommendations	Educational institutions and policymakers in the United States	‐ Education aspect is recognized as crucial to increase HCP competence and make the future‐proof for personalized medicine ‐ Guidelines (e.g., Association of Professors of Human and Medical Genetics) exist for HCPs and educational programs (e.g., genomics‐based education within the USUHS School of Medicine and the USUHS Graduate School of Nursing) are on offer for students
Westphalen 2022^7^	DKH consensus statement	HCPs involved in treatment decisions	‐ Consensus statements covering different aspects of precision cancer medicine and its implementation into clinical practice ‐ Physicians need to stay up‐to‐date with cBT use in precision oncology, to correctly decide on the best treatments for their patients. Medical education programs must be improved to include cBT‐related and precision oncology concepts ‐ Collaboration between treating physicians and molecular pathologists is key to provide access to state‐of‐the‐art cBT ‐ Reporting of alterations identified by cBT should be harmonized across cancer centers in Germany (through, e.g., the use of harmonized reporting algorithms) ‐ Clinical implications of cBT results should be decided in multidisciplinary teams, preferably molecular tumor boards ‐ Molecular tumor boards should meet universal personnel requirements (molecular pathologist, treating specialist oncologist, an oncologist from a different field and ideally also genetics, molecular biology and bioinformatics experts) to address cBT‐related complexities and determine best patient management strategies ‐ To facilitate access to cBT‐informed therapies, it is advised to concentrate the cBT efforts in specialized (academic) centers and create a network of centers for conduct of clinical trials for patients with rare alterations. This concentration should also facilitate reimbursement modalities for all cBT‐related aspects ‐ Patient data should be collected in structured and harmonized databases with appropriate bioinformatics support ‐ cBT should be integrated into holistic and adequate counseling by the treating oncologist and other relevant experts (e.g., human geneticists and psycho‐oncologists)
The Cancer Genome Atlas^59^	Online database	Oncology and genomics researchers	‐ Database of matched (somatic and germline) variants identified in cancer, growing in real time and accessible for researchers and clinicians worldwide

Abbreviations: ACMG, American College of Medical Genetics and Genomics; AMP, Association for Molecular Pathology; ASCO, American Society of Clinical Oncology; CAP, College of American Pathologists; cBT, comprehensive biomarker testing; CSER, Clinical Sequencing Exploratory Research; DKH, Deutsche Krebshilfe (engl. German Cancer Aid); DoD, Department of Defense; eMERGE, Electronic Medical Records and Genomics; HCP, healthcare professional; MHS, Military Health System; NGS, next‐generation sequencing; USUHS, Uniformed Services University of the Health Sciences.

While many HCPs acknowledged that keeping up with advancements in precision oncology (e.g., up‐to‐date scientific literature) was important,^45^ this was found to additionally burden the HCPs^20^ and require support from a multidisciplinary team.^7^, ^11^, ^41^, ^55^

Aside from the key role of genetics HCPs,^44^, ^45^, ^53^, ^55^, ^61^ the roles of non‐oncology HCPs were not extensively discussed in the identified studies. Integrating cBT into daily clinical practice will require oncologists to collaborate with other professions such as navigators, oncology nurses, geneticists, bioinformaticians, pathologists, laboratory personnel, and psychologists.^7^, ^55^ As exemplified by a single‐center study from Saudi Arabia, a nurse coordinator may support the treating oncologists by, for example, guiding the patients in their trajectory and coordinating patient referrals and cBT.^62^ The roles of pathology experts and non‐specialist HCPs in supporting their patients during cBT‐related decision‐making were also suggested.^52^ Finally, primary care providers (general practitioners and nurses) can help advocate for patients' goals within wider multidisciplinary teams, support patients' self‐management and improve patient outcomes.^52^, ^61^

Main findings and recommendations of this review are summarized in Table 5.

**TABLE 5:** Summary of main manuscript themes and messages.

Theme	Messages
Characteristics and processes of patient‐HCP communication	‐ High interest of patients and public, but generally low understanding due to complexity of cBT‐related information and the uncertainty inherent to cancer (e.g., of an individual benefit) ‐ Patients' expectations from precision oncology approaches are high: individualized treatment, high likelihood of therapeutic benefit/cure ‐ Conflicting and changing patients' information preferences due to new knowledge and concern for family members ‐ There is a need to distinguish between clinical care and clinical trial context: expected versus realistic benefit versus no benefit. The interweaving of research and clinical care poses a significant challenge for the communication of the impact (benefit) for the individual patient ‐ It is difficult for HCPs to combat challenges posed by patients: time and effort needed to explain all details, lack of own knowledge and confidence, concern for how this may influence patients' socio‐economic status (e.g., finances, insurance, and working situation) and overall health ‐ It is difficult for HCPs to communicate about secondary findings and VUSs, so sometimes high emphasis is placed on actionable alterations; resulting in the need to temper the wording to not bias patients' expectations or their understanding of the condition
Mechanisms to support shared decision‐making in precision oncology	‐ Patients' high interest and motivation are confounded by their hindered understanding of cBT‐related concepts and potentially also the influence of their family and/or caregivers on their decision‐making ‐ Value of physician to provide relevant information as a trusted and empathic source and possibility to influence patients' decision‐making regarding their cancer journey
Perspectives of HCPs	‐ HCPs must find ways to facilitate patients' comprehension but are faced with challenges such as the lack of time, resources, patient‐focused materials and lack of knowledge and formal training in genomics ‐ Some guidelines and educational programs have been established to help HCPs in cBT‐related communication, but more effort is needed in this area ‐ Recommendation for general practitioners, nurses and non‐oncology specialists familiar with the patient's health status to support oncologists in the management of cancer patients ‐ Recommendation for oncologists to function in a multidisciplinary team with oncology nurses, geneticists, bioinformaticians, pathologists, laboratory personnel and psychologists to provide optimal care for their patients ‐ Evidence of non‐oncologist profiles in cBT‐oriented communication is limited

Abbreviations: cBT, comprehensive biomarker testing; HCP, healthcare professional; VUS, variants of uncertain significance.

## 4 CONCLUSIONS

Given cBT's promise for precision oncology, effective cBT‐related communication is key to ensure proper patient understanding, manage the inherent uncertainties, provide psychological support, and allow for informed shared decision‐making.^11^, ^12^, ^19^, ^36^, ^41^, ^45^ We found that patients' needs regarding information on cBT interact with their expectations and their understanding of implications of testing. Difficulties in understanding cBT‐related information, the uncertainty of a potential benefit, and the handling of incidental findings are challenging. Trust toward the treating physician seems to play an important role regarding shared decision‐making. Moreover, additional information materials and decision support tools as well as the integration of caregivers need to be further elaborated. Finally, a lack of continuing education opportunities for HCPs became apparent. The findings of this scoping review have significant implications for communication, information, and patient management in the context of precision oncology.

The complexity in cBT is high and may lead to a perceived overload of information or misconceptions.^20^, ^33^, ^36^ Patients need guidance throughout the cBT process and associated treatment as well as information tailored to their individual situation.^12^, ^15^, ^16^, ^33^, ^35^ Careful management of relevant information and trust in their physician can help the patients feel informed rather than overwhelmed, so HCPs should choose an appropriate manner (e.g., language, illustration, and emotional support) and amount of information to be delivered, also pre‐testing.^16^, ^37^, ^45^ To tailor information according to individual preferences, informed consent processes might be, for example, organized using a “tiered and binned model of counseling,” as previously developed for the purpose of multiplex testing for cancer susceptibility.^63^ Alternatively, established frameworks can be used to explain levels of evidence supporting the use of cBT‐guided therapies,^57^, ^58^, ^60^ so that patients can choose information on only certain types of findings (e.g., actionable mutations). Generally, shared decision‐making can be facilitated by providing patients with comprehensive and understandable information that is easily accessible and from validated sources.^20^, ^31^, ^37^, ^43^, ^64^

Information management is especially important when discussing the potential individual benefits for patients participating in cBT, as we found frequent mismatches between high patients' expectations and actual treatment benefits.^20^, ^21^, ^37^ Considering the interweaving of clinical care and clinical research, the lack of clarity about the expected benefits of cBT may exacerbate patients' cancer‐related distress.^11^, ^19^, ^20^, ^41^, ^65^ Therefore, addressing various aspects of cBT‐related uncertainty seems crucial.^65^, ^66^ Previous work showed that appraisal rather than avoidance of uncertainty allows for active coping^67^, ^68^, ^69^ and shared decision‐making.^69^ A caring relationship, communicating continuity of care regardless of cBT results and ensuring support can help patients deal with uncertainty.^68^, ^69^, ^70^ Engagement and empowerment of patients can be supported by multidisciplinary teams.^71^

Equally important, the possibility of receiving secondary/germline/incidental findings and the related communication are critical.^17^, ^21^, ^26^, ^37^, ^72^ Given the potential impact for family members and caregivers, patients' preferences for involving family members need to be assessed^17^, ^26^, ^30^, ^37^ to develop harmonized communication processes. Communication and decision aids might support both HCPs and patients.^73^ Besides, there is a need for communication training of physicians dealing with these issues and identifying patients with an additional need for psychological support.^74^ While some guidelines and resources are available to HCPs to guide and improve their cBT‐related communication, these seem to be limited to English language records and focus on high‐income‐countries,^7^, ^55^, ^57^, ^58^, ^59^, ^60^, ^61^ indicating a need for more research and expert debates to involve also non‐English‐speaking geographical area and low‐ and middle‐income countries. Moreover, well‐known concepts and guidelines of genetic/genomic counseling, precision medicine, social and behavioral science should be integrated and adapted to the context of cBT in cancer patients based on multidisciplinary consensus.^75^

Further, structures need to be adapted and/or developed to support adequate patient management. For example, the implementation of specific consultation hours for patients to discuss with cBT specialists (e.g., genetics HCP) might support HCPs to combat the challenges associated with cBT‐related communication. These structures should enable the necessary collaboration with multiprofessional teams and a continuing information flow for the patient. Certainly, specialists of psycho‐oncology, ethics, and palliative care should be involved in the development of this coordinated process,^10^, ^69^, ^75^ especially for challenging decisions (e.g., continuing treatment versus end‐of‐life and advanced care planning).^76^, ^77^

However, HCPs frequently felt they had insufficient time, knowledge, and expertise to effectively communicate about cBT implications and outcomes to their patients.^20^, ^46^, ^47^, ^48^, ^49^ This corroborates the previously noted lack of HCPs' confidence in cBT approaches and results disclosure.^78^ Information on the level of HCPs' cBT‐related knowledge was derived only from self‐assessments, emphasizing the need for HCPs' knowledge to be also objectively measured in future studies. Furthermore, some oncologists were faced with a lack of up‐to‐date scientific materials and higher barriers to cBT uptake by patients and their families.^49^, ^50^ Importantly, the number of studies dealing with HCP‐specific barriers to cBT‐related communication was low, clearly indicating that more research is needed and reinforcing the point on suitable and continuing HCPs' education in this area.^49^, ^50^ Importantly, disseminating cBT‐related knowledge among HCPs and improving uptake of cBT across institutions must be guided by principles of equity and patient‐focused practice.^79^

This study has several limitations. We cannot exclude the possibility that relevant records were omitted because some search terms were broad and only PubMed was searched. It was difficult to define appropriate search strings due to varied and inconsistent terminology and lack of appropriate MeSH terms used in the field. Also, the necessity to include broad terms such as “communication” increased the number of irrelevant articles identified in PubMed. We recommend that future studies use harmonized terminology in their titles and abstracts as well as define and use consistent MeSH terms, to facilitate identification of relevant studies via structured PubMed searches. Additionally, the included studies frequently missed quantitative data (e.g., percentage of participants reporting an outcome or agreeing with statements), had heterogeneous designs, used cBT‐related terms interchangeably and lacked distinctions between testing and finding types.

The review strengths were the extensive and systematic approach to search strategy, the breadth of covered themes and a strong focus on HCPs' needs and roles in patient‐focused communication of cBT‐related concepts. A notable strength was also the versatile expertise of the authors (physicians, oncologists, nursing specialists, researchers in oncology and psycho‐oncology, clinical psychologists, patients, patient representatives, patient engagement specialists).

In conclusion, effective patient‐HCP communication is important to ease psychosocial distress and support understanding of patients when results are disclosed. Advanced genomics education of HCPs facing oncology patients is necessary to facilitate this communication. Knowledgeable and confident HCPs will be able to empower patients and secure support of their families and caregivers by conveying relevant and up‐to‐date cBT knowledge and efficiently guiding shared decision‐making in precision oncology.

## AUTHOR CONTRIBUTIONS

**Theresia Pichler:** Conceptualization (equal); data curation (equal); formal analysis (equal); investigation (equal); methodology (equal); supervision (equal); writing – original draft (equal); writing – review and editing (equal). **Friederike Mumm:** Conceptualization (equal); investigation (equal); methodology (equal); supervision (equal); writing – original draft (equal); writing – review and editing (equal). **Navdeep Dehar:** Conceptualization (equal); investigation (equal); methodology (equal); supervision (equal); writing – original draft (equal); writing – review and editing (equal). **Erin Dickman:** Conceptualization (equal); investigation (equal); methodology (equal); supervision (equal); writing – original draft (equal); writing – review and editing (equal). **Celia Díez de Los Ríos de la Serna:** Conceptualization (equal); investigation (equal); methodology (equal); supervision (equal); writing – original draft (equal); writing – review and editing (equal). **Andreas Dinkel:** Conceptualization (equal); investigation (equal); methodology (equal); supervision (equal); writing – original draft (equal); writing – review and editing (equal). **Kathrin Heinrich:** Conceptualization (equal); investigation (equal); methodology (equal); supervision (equal); writing – original draft (equal); writing – review and editing (equal). **Merel Hennink:** Conceptualization (equal); investigation (equal); methodology (equal); supervision (equal); writing – original draft (equal); writing – review and editing (equal). **Anndra D. Parviainen:** Conceptualization (equal); investigation (equal); methodology (equal); supervision (equal); writing – original draft (equal); writing – review and editing (equal). **Vincent Raske:** Conceptualization (equal); data curation (equal); formal analysis (equal); investigation (equal); methodology (equal); supervision (equal); writing – original draft (equal); writing – review and editing (equal). **Nicole Wicki:** Conceptualization (equal); data curation (equal); formal analysis (equal); investigation (equal); methodology (equal); supervision (equal); writing – original draft (equal); writing – review and editing (equal). **Amy C. Moore:** Conceptualization (equal); investigation (equal); methodology (equal); supervision (equal); writing – original draft (equal); writing – review and editing (equal).

## FUNDING INFORMATION

Teleconferences and meetings for manuscript development were organized by the From Testing to Targeted Treatments (FT3) Program. The authors did not receive payment for their contribution to the development of the manuscript. Further information about the governance structure of the FT3 Program (including funding, membership and the decision‐making process of the organization) is available at: https://www.fromtestingtotargetedtreatments.org/governance/. Medical writing and coordination support was provided by Akkodis Belgium and funded by the FT3 Program.

## CONFLICT OF INTEREST STATEMENT

Theresia Pichler, Friederike Mumm, and Amy C. Moore received support from the FT3 program for project coordination. Theresia Pichler received support from the Comprehensive Cancer Center Munich for attending meetings and travels. Friederike Mumm is member of the BZKF AG “Shared Decision‐Making,” co‐chair of the DGHO working group “Psycho‐Oncology,” chair of the TZM working group Psycho‐oncology and N‐PSOM and mandate of the S3 guidelines DGHO & pso (supportive care, suicidality, cervical carcinoma, follicular lymphoma), founding member of the clinical Ethics Committee at the LMU University Hospital of Munich, and joint responsible of Patientenorienierte Kommunikation (POK) and KommMeCuM at the Medical Faculty of LMU Munich; she is a consortium partner of Transsektorales personalisiertes Versorgungskonzept für Patienten mit seltenen Krebserkrankungen (TARGET), funded by the GBA‐Innovationsfond. Erin Dickman participated on a Precision Medicine Advisory Board—Cancer Support Community. Andreas Dinkel received a research grant from the German Innovation Fund and honoraria from Gilead Sciences; he is also a Board Member of the German Association for Psycho‐Oncology (PSO) and Vice‐Speaker of the Working Group for Psycho‐Oncology of the German Comprehensive Cancer Center Network. Kathrin Heinrich received consulting fees from Servier, honoraria for presentations from Roche and Taiho, and support for attending meetings and travel from Servier, Merck and Amgen; she is also Board Member of the Arbeitsgemeinschaft Internistische Onkologie. Amy C. Moore participated in data safety monitoring/advisory boards from Amgen, AstraZeneca, Bayer, Daiichi Sankyo, Exact Sciences, Gilead, Jazz Pharmaceuticals, and Novartis and has a leadership role in the NTRKers Board of Directors and is an employee of LUNGevity. Vincent Raske and Nicole Wicki are employees of The Synergist, a non‐profit organization, which includes the industry‐sponsored FT3 Program. Nicole Wicki is also program director for the FT3 program. Vincent Raske is a program manager for the FT3 program and general secretary of The Young Republic. Navdeep Dehar, Merel Hennink, Celia Díez de Los Ríos de la Serna, and Anndra D. Parviainen declare no conflict of interest.

## Supporting information


Table S1.

## Data Availability

Data sharing is not applicable—no new data generated.
