# Supplementary material for: Understanding communication between patients and healthcare professionals regarding comprehensive biomarker testing in precision oncology: A scoping review
Source: Cancer Med. 2024 Jan 31;13(3):e6913. doi: 10.1002/cam4.6913 (PMC10905543; doi:10.1002/cam4.6913)
Supplement: Supplementary file 1 — Table S1. [file CAM4-13-e6913-s001.docx]

**Understanding communication between patients and healthcare professionals regarding comprehensive biomarker testing in precision oncology: a scoping review**

Theresia Pichler, Friederike Mumm, Navdeep Dehar, Erin Dickman, Celia Díez de Los Ríos de la Serna, Andreas Dinkel, Kathrin Heinrich, Merel Hennink, Anndra D. Parviainen, Vincent Raske, Nicole Wicki, Amy C. Moore

***Supplementary material***

**Supplementary table 1: Characteristics of included studies**

| **Authors** | **Source** | **Year** | **Publication type** | **Study WHO region** | **Study country** | **Study year(s)** | **Audience** | **Cancer type** | **Study design** | **Identified by** |
| --- | --- | --- | --- | --- | --- | --- | --- | --- | --- | --- |
| Roberts JS MI-ONCOSEQ Study team [31] | Cancer Med | 2019 | Original research | AMR | USA | 2014–2016 | Oncologists, Patients | Advanced stage solid tumor malignancies | Prospective survey | PubMed search |
| Bylstra Y [34] | Hum Genomics | 2017 | Original research | WPR | Singapore | 2015 | HCPs, Researchers | Breast cancer | Prospective survey | PubMed search |
| Mamzer MF [39] | J Transl Med | 2017 | Original research | EUR | France | 2013–2015 | HCPs, Cancer patients | Not specified | Prospective | PubMed search |
| Pinheiro APM [30] | Cancer | 2017 | Original research | AMR | USA | Not specified | HCPs, Cancer researchers, Caregivers | Lung, colorectal, breast, melanoma, gastric, liver, other cancer | Prospective survey | Expert referral |
| Al-Kaiyat MO [62] | Clin J Oncol Nurs | 2018 | Brief report | EMR | Saudi Arabia | Not specified | HCPs | Lung cancer | Case report | PubMed search |
| Bijlsma RM [26] | Fam Cancer | 2018 | Original research | EUR | The Netherlands | 2014 | Oncologists | Brain tumor, breast cancer, cholangiocarcinoma, colon cancer, epithelioid hemangio endothelioma, larynx cancer, melanoma, ovarian cancer, pancreatic cancer, prostate cancer, renal-cell cancer, testicular cancer | Observational prospective study | PubMed search |
| Bijlsma RM [17] | Psychooncology | 2018 | Original research | EUR | The Netherlands | 2014 | HCPs, Oncologists, Oncology nurses | Brain tumor, breast cancer, cholangiocarcinoma, colon carcinoma, epithelioid hemangio endothelioma, larynx carcinoma, melanoma, ovarian cancer, pancreatic cancer, prostate cancer, renal-cell carcinoma, testicular cancer | Prospective survey | PubMed search |
| Gray SW [54] | J Am Med Inform Assoc | 2018 | Original research | AMR | USA | 2016 | Oncologists, Surgeons | Metastatic breast and lung cancer, metastatic melanoma | Randomized, vignette-based survey study | PubMed search |
| Halbert CH [40] | Am J Med Genet C Semin Med Genet | 2018 | Original research | AMR | USA | N/A | HCPs, Oncologists, Genetic counselors | Not specified; mentions breast cancer | Expert opinion | PubMed search |
| Kaphingst KA [38] | Transl Behav Med | 2018 | Original research | AMR | USA | 2005–end not specified | HCPs, Oncologists, Geneticists, Patients | Breast cancer | Prospective survey | PubMed search |
| Krauss JC [50] | JCO Clin Cancer Inform | 2018 | Original research | AMR | USA | 2014–2015 | HCPs, Cancer patients | Gastro-intestinal cancers | Prospective study | PubMed search |
| O'Neill SC [41] | J Health Commun | 2018 | Original research | AMR | USA | 2013–2015 | Oncologists | Breast cancer | Prospective survey | PubMed search |
| Peterson EB [43] | Transl Behav Med | 2018 | Systematic review | AMR | USA | 2010–2017 | HCPs, Genetic specialists (i.e., genetic counselors and clinical geneticists) | Not specified, but including breast, colorectal, ovarian, extracolonic, lung, prostate, esophageal, skin cancer, familial melanoma, Lynch syndrome | Systematic review and meta-analysis | PubMed search |
| Pujol P [42] | Eur J Hum Genet | 2018 | Guideline | EUR | France | 2016–2017 | HCPs, Oncologists, Cancer patients, Regulators | Not specified (excluding pediatric cancers) | Expert consensus | PubMed search |
| Stoeklé H [11] | Sci Eng Ethics | 2018 | Guideline | EUR | France | N/A | Bioethics experts, Patients, Clinicians, Researchers in private and public sector, Institutes and companies focusing on biobanks, databases, bioinformatic platforms and genomic platforms | Not specified | N/A | PubMed search |
| de Hosson L [51] | Orphanet J Rare Dis | 2019 | Original research | EUR | The Netherlands | 2015–2016 | Patients, Oncology nurses, Medical oncologists | Neuroendocrine tumors | Randomized controlled trial | PubMed search |
| Horgan D [35] | Public Health Genomics | 2019 | Expert Opinion | EUR | Belgium; the Netherlands | 2019 | HCPs, Patients | Not specified | Survey | PubMed search |
| Kaphingst KA [28] | Genet Med | 2019 | Original research | AMR | USA (and one person affiliated in Singapore) | 2010–2017 | Oncologists, Genetic counselors, Cancer patients | Not specified (including breast and colorectal cancer) | Scoping review | PubMed search |
| Vetsch J [44] | Semin Oncol | 2019 | Systematic review | WPR | Australia | 2001–2018 | HCPs | Not specified | N/A | Expert referral |
| Yip S [55] | Curr Oncol | 2019 | Guideline | AMR | Canada | 2019 | Oncologists, HCPs | Not specified | Consensus-based guideline | PubMed search |
| Bartley N [32] | J Med Genet | 2020 | Original research | WPR | Australia | 2017–2018 | HCPs, Cancer patients | Gastro-intestinal, breast, genito-urinary, blood, central nervous system, sarcoma, head and neck, other cancer | Prospective survey | Expert referral |
| Waltz M [22] | J Genet Couns | 2020 | Brief report | AMR | USA | 2012–2017 (findings that are part of a larger study, NCGENES) | Research clinicians, GPs, Oncologists working on breast cancer, Women with or at risk of developing breast cancer | Breast and ovarian cancer | Secondary analysis of data collected in another study | PubMed search |
| Wolyniec K [36] | Psycho oncology | 2020 | Systematic review | WPR | Australia | 2007–2017 | HCPs, Oncologists, Oncology nurses, Psychologists | Not specified (including ovarian, gastro-intestinal, breast, lung cancer, melanoma) | N/A | PubMed search |
| Yabroff KR [47] | J Natl Cancer Inst | 2020 | Original research | AMR | USA | 2017 | Oncologists, Cancer patients | Not specified | Prospective survey | PubMed search |
| Buckenmaier SS [49] | J Cancer Educ | 2021 | Original research | AMR | USA | 2017 | HCPs, Oncologists, Oncology nurses, Multi-marker tumor panel test developers | Solid tumors, hematologic cancers | Prospective survey | PubMed search |
| Bunnik EM [37] | Eur J Cancer | 2021 | Expert Opinion | EUR | The Netherlands | N/A | Oncologists, Clinical geneticists, Genetic counselors | Not specified (including cancers for which genomic testing is standard practice) | N/A | PubMed search |
| Doll B [61] | Mil Med | 2021 | Review | AMR | USA | N/A | Clinicians | Not specified | N/A | PubMed search |
| Giri VN [80] | JCO Precis Oncol | 2021 | Original research | AMR | USA | 2019 | HCPs, Patients, General public | Not specified | Prospective survey | PubMed search |
| Hall MJ [45] | Public Health Genomics | 2021 | Original research | AMR | USA | 2019–2020 | HCPs, Educators, Regulators | Not specified | Prospective survey | PubMed search |
| Hamilton JG [20] | Per Med | 2021 | Original research | AMR | USA | 2019 | Oncologists | Breast cancer, melanoma, thoracic cancer | Prospective survey of focus groups | PubMed search |
| Koczwara B [52] | Aust J Gen Pract | 2021 | Guideline | WPR | Australia | N/A | HCPs, GPs, Nurses | Not specified | N/A | PubMed search |
| O'Neill SC [46] | Patient Educ Couns | 2021 | Original research | AMR | USA | 2017–2018 | HCPs, Cancer patients | Breast cancer | Prospective survey | PubMed search |
| Pichler T [12] | Support Care Cancer | 2021 | Original research | EUR | Germany | 2017–2018 | HCPs, Cancer patients | Gastro-intestinal tumor, urological cancer, neuroendocrine tumor, head and neck tumor, breast tumor, melanoma, sarcoma, gynecological tumor, lung tumor, lymphoma, brain tumor | Prospective survey | PubMed search |
| Spees LP [48] | Patient Educ Couns | 2021 | Original research | AMR | USA | 2017 | HCPs, Researchers, Cancer patients | Solid tumors, hematologic malignancies | Post-hoc analysis | Expert referral |
| Bartley N [19] | Patient Educ Couns | 2022 | Original research | WPR | Australia | 2017–2019 | HCPs, Cancer patients | Advanced or metastatic solid tumors | Prospective survey | PubMed search |
| Best MC [33] | Support Care Cancer | 2022 | Original research | WPR | Australia | 2017–2019 | HCPs, Cancer patients | Advanced cancer with last-line therapy/exhausted treatment options | Prospective survey | Expert referral |
| Bradbury AR [27] | Cancer | 2022 | Original research | AMR | USA | 2016–2019 | HCPs, Cancer patients | Advanced cancer | Prospective cohort study | Expert referral |
| Dehar N [18] | Curr Oncol | 2022 | Original research | AMR | Canada | 2019 | HCPs | Breast, colon, pancreas, renal, melanoma, lung, prostate cancer | Prospective survey | Expert referral |
| Meiser B [29] | Eur J Med Genet | 2022 | Original research | WPR | Australia | Not specified | HCPs, Cancer patients | Not specified | Prospective survey | Expert referral |
| Petrillo LA [21] | Cancer | 2022 | Original research | AMR | USA | 2018–2020 | HCPs, Cancer patients | Lung cancer | Prospective survey | PubMed search |
| Shimada S [53] | Eur J Hum Genet | 2022 | Original research | WPR | Japan | 2020 | HCPs, Cancer patients | Not specified | Prospective survey | PubMed search |
| Westphalen BC [7] | Eur J Cancer | 2020 | Practice guideline | EUR | Germany | Not specified | HCPs | Not specified | Prospective questionnaire with Delphi approach consensus | Expert referral |

| AMR, EMR, EUR, WPR, World regions defined by the World Health Organization (WHO), Region of the Americas, Eastern Mediterranean region, European region, West-Pacific region; GP, general practitioner; HCPs, healthcare professionals; N/A, not applicable; USA, United States of America. |
| --- |
